# A Novel Extrusion for Manufacturing TiBw/Ti6Al4V Composite Tubes with a Quasi-Continuous Reinforced Structure

**DOI:** 10.3390/ma10040375

**Published:** 2017-03-31

**Authors:** Jianlei Yang, Xueyan Jiao, Wenzhen Chen, Wencong Zhang, Guofeng Wang

**Affiliations:** School of Materials Science and Engineering, Harbin Institute of Technology, Harbin 150001, China; jlyhit@163.com (J.Y.); hit105@163.com (X.J.); wczhang@hit.edu.cn (W.Z.); gfwang@hit.edu.cn (G.W.)

**Keywords:** titanium matrix composites, tube, hot extrusion, microstructure, mechanical performance

## Abstract

The present work introduces a novel extrusion with filler material to produce the high-performance TiBw/Ti-6Al-4V composite tube with a quasi-continuous reinforced structure. A simulation was adopted to study the effect of the filler material on the shape accuracy of the tubes. Based on the simulation results, the flow stress of the filler material was not the important factor, but the friction coefficient between the filler and TiBw/Ti-6Al-4V composite and the canning shape were critical to the tube precision. The microstructure and mechanical performance for the as-extruded TiBw/Ti-6Al-4V composite tubes were systematically investigated. After extrusion, the transverse section microstructure of the TiBw/Ti6Al4V composite tube remained quasi-continuous and the TiBw were rotated to align the extrusion direction. Moreover, the tensile strength and elongation reached 1240 MPa and 13.5%, resulting from dynamic recrystallization and whisker rotation.

## 1. Introduction

In the past decades, due to high special strength, high special stiffness, excellent wear resistance and high temperature durability, titanium matrix composites (TMCs) have received considerable attention as the optimal candidate material for a range of commercial automotive, aerospace and military applications [1,2,3,4]. As one kind of TMC, discontinuously reinforced titanium matrix composites (DRTMCs), fabricated by in situ methods, are sought-after for their superior and isotropic properties along with their low cost [5,6,7]. In practice, TiB whiskers (TiBw) are regarded as the optimal reinforcement due to their high modulus, hardness, good chemical compatibility and high thermal expansion coefficient with the Ti matrix [8,9,10,11,12,13,14]. However, the TMCs prepared by the conventional powder metallurgy (P/M) method generally have inferior performance compared with those obtained by the casting technique in view of the plasticity at room temperature [2,15,16,17]. In our previous works [9,18,19], it was well demonstrated that tailoring a novel network reinforcement distribution of TiBw/Ti6Al4V composites could avoid the serious drawbacks.

Undoubtedly, network-structured TiBw/Ti6Al4V composites are a preferable material for tubes applied in hydraulic pipelines for the aerospace, nuclear power and auto industries. The conventional production processes for tubes, such as skew rolling [20], spinning [21], extrusion [22], etc., can realize volume production. Unfortunately, most of the seamless titanium alloy tubes are made of pure titanium or low-strength titanium alloy [23,24], which has confined their wide application in the last decade. There is no doubt that a higher strength and modulus would bring more difficulties to the deformation of the titanium matrix composites (TMCs). Thus, it is necessary to explore a novel process to produce TiBw/Ti6Al4V composite tubes. Inspired by traditional canning extrusion [9,25], hot extrusion with a filler material was attempted to produce the TiBw/Ti6Al4V composite tubes. Compared with the conventional extrusion process, this process has the advantage in the fabrication of thin tubes with a large length-diameter ratio, which skips the requirement of the rigid spindle.

In this paper, TiBw/Ti6Al4V composite billets were prepared by low-energy milling and hot-pressed sintering. In addition, the composite tubes were fabricated by hot extrusion with filler material. In addition, numerical modeling was used to guide the selection of the filler material and canning shape. Furthermore, the microstructure and mechanical properties of the TiBw/Ti6Al4V composite tube were also investigated in detail.

## 2. Experimental Section

**Figure 1:**
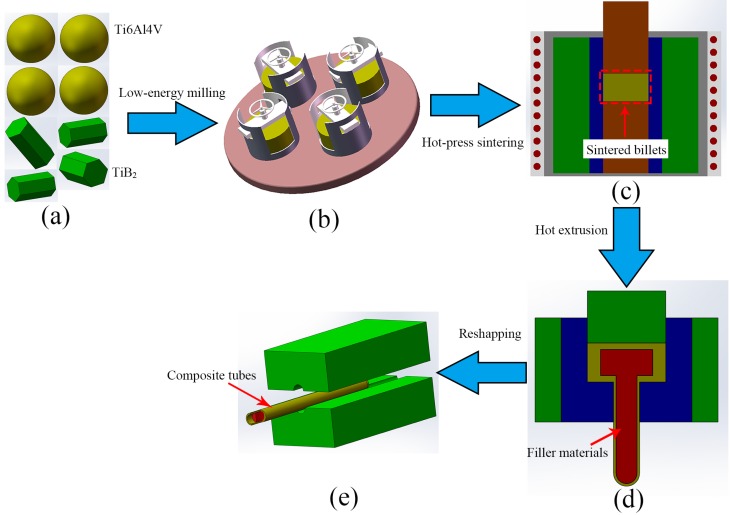
Schematic illustration of procedures to fabricate TiBw/Ti6Al4V composite tube: (**a**) Ti6Al4V and TiB_2_ powders; (**b**) the low-energy milling; (**c**) the sintering process; (**d**) the extrusion process; (**e**) the reshaping process.

The spherical Ti6Al4V powders (110 μm) and prismatic TiB_2_ (2 μm) powders were selected as the starting materials. The chemical composition (wt. %) of the Ti6Al4V powders purchased from Shanxi xiyu Metallic Materials Ltd. (Shanxi, China) are listed in Table 1. The purity of the TiB_2_ powders provided by Zibo special ceramics Ltd, PR China is higher than 98.6%. Figure 1 showed the schematic illustration of procedures to fabricate TiBw/Ti6Al4V composite tubes. The Ti6Al4V powders and TiB_2_ powders were low-energy milled in planetary ball mill (QM-3SP4) with a ball-to-powder weight ratio of 5:1 at 200 rpm for 8.0 h under the argon protection. Next, the blended powders were sintered at 1200 °C for 1.0 h under a pressure of 20 MPa in high vacuum (10^−2^ Pa) followed by furnace cooling. Thirdly, the sintered billets were machined with the blind hole and filled with the filler materials, then performed hot extrusion and reshaping process.

**Figure 2:**
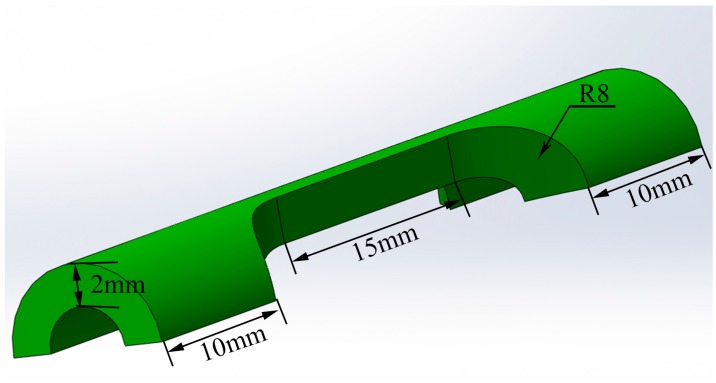
Dimensioned schematic of the tubes tensile specimen.

Specimens for microstructural investigation and tensile test were directly cut from the composites tubes. The microstructural samples were then prepared using the conventional techniques of grinding, polishing and subsequent etching using the Kroll’s solution (5 vol % HF + 10 vol % HNO_3_ + 85 vol % H_2_O) for 10 s and observed by scanning electron microscope (SEM, Hitachi S-570) and optical microscope (OM, OLYMPUS GX71). Tensile tests were conducted using an Instron-5569 at room temperature and Instron-5500R at 600 °C with a constant crosshead speed of 0.9 mm/min. It should be noted that the tensile specimens were held at 600 °C for 3 min prior to the addition of load. Dog-bone specimens have gauge dimensions of 15 mm × 2 mm × 2 mm (shown in Figure 2) and a total of three specimens were tested for each material.

## 3. Results and Discussion

### 3.1. Numerical Modeling

In order to optimize the selection of the filler material and the canning shape of the tubes, the extrusion process of TiBw/Ti6Al4V composite tubes was simulated by the DEFORM-2D software. It must be noted that the constitutive model of this material was obtained by the hot compression reported in our previous work [26]. The other material parameters needed in finite element modeling are listed in Table 2. Among them, the flow stress *σ* of the filler material, the frictional coefficient *f* between the filler and canning and the bottom thickness *t* of the canning were regarded as the research parameters.

**Figure 3:**
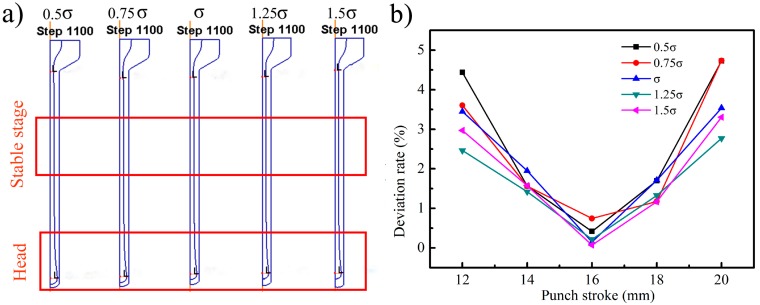
The simulation result of extrusion process of TiBw/Ti6Al4V composite tube with the different filler materials: (**a**) the extrusion result; (**b**) deviation rate of thickness.

In order to ensure the whole simulation process and obtain the single impact of the flow stress, the frictional coefficient *f* between the filler and canning and the bottom thickness *t* were supposed to be 0.7 and 3.0 mm, respectively. The flow stress of the TiBw/Ti6Al4V composite was presented as *σ*, and the flow stress multiplied by a series of coefficients (0.5, 0.75, 1, 1.25, 1.5) was defined as 0.5*σ*, 0.75*σ*, *σ*, 1.25*σ*, 1.5*σ*. Figure 3 showed the numerical simulation results of the filler materials with different flow stresses subjected to hot extrusion. It can be seen that the “Big Head” phenomenon appeared on the head and the radius of the filler materials gradually decreased until entering into the stable extrusion stage. The deviation rate from the average of the inner diameter for the extruded tubes in the stable stage was calculated and it was no more than 5.0%. In addition, the value of the maximum radius decreased slightly with the flow stress increasing.

**Figure 4:**
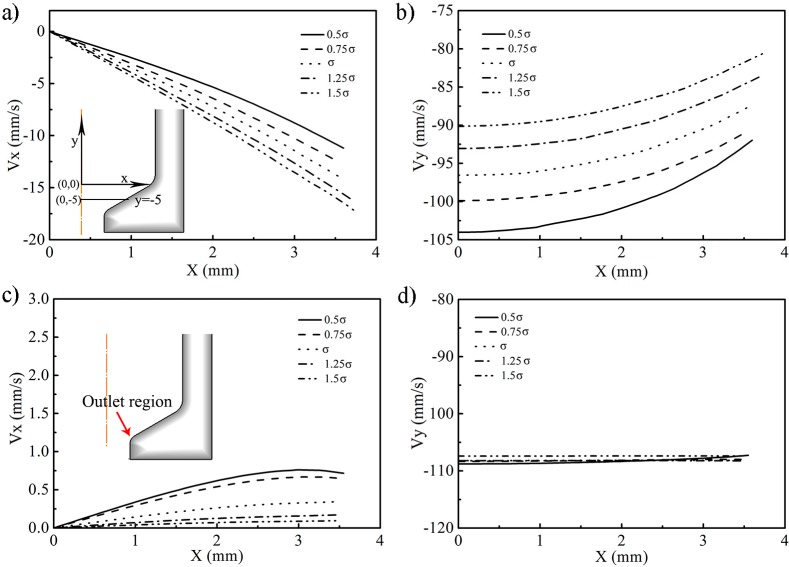
Flow velocity of the different filler materials at horizontal line *y* = −5 mm (**a**) X axis direction; (**b**) Y axis direction; and outlet region (**c**) X axis direction; (**d**) Y axis direction.

Figure 4 showed the flow velocities of different filler materials at the deformation region (the horizontal line *y* = −5 mm) and outlet region with the punch stoke at 16 mm. As can be seen from Figure 4a, at horizontal line *y* = −5 mm in the deformation region, the velocity along the X axis was relatively lower with a lower deformation resistance for the filler materials. However, the filler material with a lower deformation resistance has a larger velocity value along the Y axis shown in Figure 4b and the maximum difference value was 13 mm/s. At the outlet region shown in Figure 4c,d, the dominated flow velocity direction was minus along the Y axis, and the speed value for different filler materials was similar. The velocity along the X axis was less than 0.75 mm/s. According to these analyses, the influence of the deformation resistance on the flow speed at the outlet region was unapparent, and this results in the similar diameter of the extruded filler materials.

**Figure 5:**
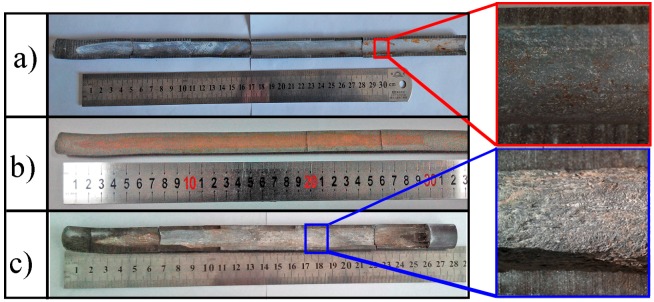
The cross-section of the extruded composite tubes with different filler materials: (**a**) 45 steel; (**b**) copper; (**c**) BN powder.

Based on the above analysis, the deformation resistance of the filler material may be an unimportant factor for the shape of the extruded tubes. In order to verify the simulated result, the extrusion process of TiBw/Ti6Al4V composite tube with different filler materials was carried out and the C45 steel, pure copper and boron nitride (BN) powder, which exhibited different flow stresses, were selected as the filler materials. The experimental results are shown in Figure 5. In particular, the C45 steel and copper are metal materials while the BN powder is a non-metallic material. It could be found that the wall thicknesses of the extruded tubes at the stable stage were uniform no matter what filler material was selected, which were agreed with the simulated results. However, as the filler material, the metal materials were difficult to remove. Thus, the non-metal materials were taken into account due to the fact that particle separation is conducive to mechanical removal. Considering the difficulty of removing the filler material, the BN powder was more appropriate as the filler material in this study.

**Figure 6:**
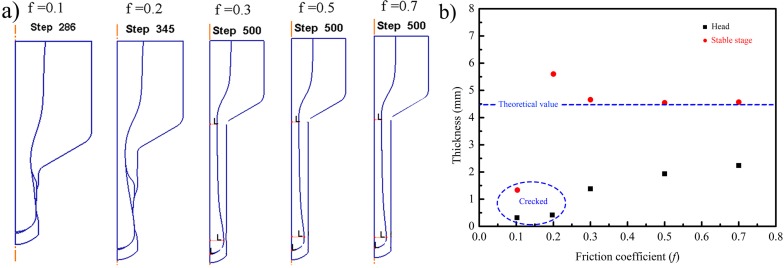
The simulation results of the extrusion process with different friction coefficients: (**a**) the simulation; (**b**) thickness of the head and stable stage.

Figure 6 demonstrated the simulation results with different friction coefficients. The friction coefficient *f* was set to be 0.1, 0.2, 0.3, 0.5, and 0.7, respectively. As shown in Figure 6a, when the friction coefficient *f* was 0.1 and 0.2, the extruded tubes were cracked around the head region. As the friction coefficient *f* reached 0.3, the crack phenomena were avoided. With the improving friction coefficient, the minimum wall thickness of the extruded tubes increased and the thickness of the stable stage tended to the theoretical value as shown in Figure 6b. The reason for the cracking at the head region can be explained by the small friction coefficient between the filler and canning which resulted in low friction resistance; thus, the larger flow velocity can be achieved. However, the flow velocity of the canning remained low due to the large friction resistance between the canning and die. The different velocities induced large tensile stress in the canning, which contributed to the wall-thickness thinning of the tubes until reaching a consistent velocity. Overall, a higher friction coefficient *f* was beneficial for the coordinated deformation between the filler and canning. In this case, the crack caused by excessive thinning can be avoided.

**Figure 7:**
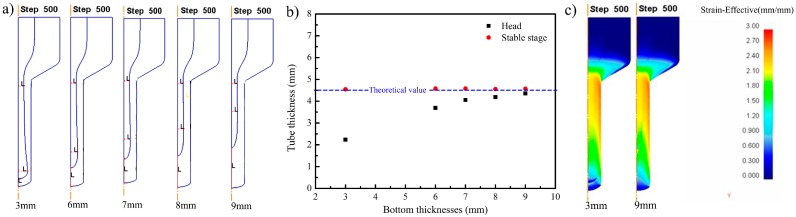
The extrusion process with different bottom thickness: (**a**) the simulation results; (**b**) thickness of different bottom thicknesses; (**c**) strain distribution maps.

Figure 7 depicted the simulation results with different bottom thicknesses of the canning. The friction coefficient between the filler and composite canning was 0.7. It could be seen that with the increased bottom thickness of the canning, the maximum filler radius in the head region decreased, which was favorable for increasing the effective length of the stable region. When the bottom thickness of the extrusion billet increased to 7 mm, the big head phenomenon disappeared and the composite tube had no wall-thickness thinning (shown in Figure 7b). As the bottom thickness continued increasing, although there was no big head phenomenon, the effective length of the stable region decreased. Figure 7c shows the strain distribution with a different bottom thickness of the canning. It was found that the severe inhomogeneous deformation was located in the head region. When the bottom thickness increased, the head region was occupied by the composite and the defects occurring in head region could be averted. On the one hand to avoid the defects, and on the other hand to get the maximum effect length of the tube, the bottom thickness of canning was selected as 7 mm.

**Figure 8:**
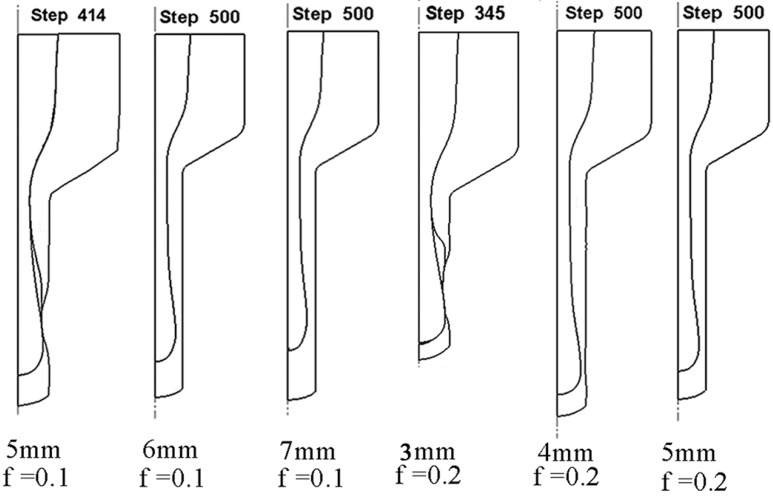
The simulation results of the extrusion process with different friction coefficient and bottom thickness.

The big head phenomenon could effectively be avoided when improving the bottom thickness of the extrusion billet. At the same time, the crack caused by excessive thinning of the tube could also be avoided. Thus the cracking defect could be avoided by coordinating the bottom thickness of the canning and the friction coefficient *f*. When the bottom thickness friction coefficient *f* was as low as 0.1 and 0.2, the corresponding limitation of the bottom thicknesses could be noticed from the simulated results shown in Figure 8. It could be seen that the lowest bottom thicknesses of the canning were 6 mm and 4 mm for the friction coefficient *f* of 0.1 and 0.2, respectively. The requirement of the bottom thicknesses decreased with the improving friction coefficient on the condition that the head region of the extruded tubes had no cracking. It could also be found that when the friction coefficient was 0.1, the head radius of the filler was larger than the stable stage and even the bottom thickness of the canning reached 7 mm, which suggested that not only increasing the bottom thickness of the canning but also improving the friction coefficient is needed to eliminate the big head phenomenon. So, the satisfactory composite tubes with a uniform distribution of the wall thickness could be achieved only by a suitable combination of the bottom thickness of the canning and the friction coefficient *f*. The simulated crack results under different bottom thicknesses of the extrusion billet and friction coefficient are shown in Table 3.

### 3.2. TiBw/Ti6Al4V Composite Tube

**Figure 9:**
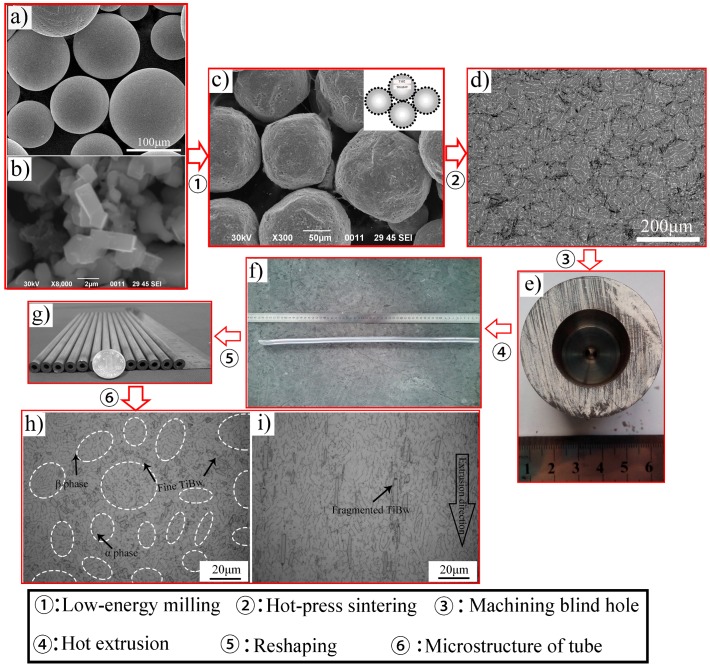
Micrographs evolution of TiBw/Ti6Al4V composite tubes: (**a**) Ti6Al4V powders; (**b**) TiB_2_ powders; (**c**) the blended powders; (**d**) the sintered microstructure; (**e**) the extruded billet; (**f**) the extruded tube; (**g**) the finished production; (**h**) the cross-section microstructure of the composite tube; (**i**) the longitudinal section microstructure of the composite tube.

Figure 9 shows the whole fabrication process of the TiBw/Ti6Al4V composite tubes from the initial powders. After low-energy milling, the fine TiB_2_ were adhered onto the surface of the large Ti6Al4V particle illustrated by the insert sketch, which is shown in Figure 9c. This distribution could provide a prerequisite condition for producing the quasi-continuous reinforced structure. During the subsequent hot-pressed sintering, TiB whiskers were in situ synthesized according to the following reaction:

Ti + TiB_2_ = 2TiB (1)

Figure 9d shows the typical composites with a quasi-continuous microstructure, which exhibited a superior combination of tensile strength and ductility compared with the composites with a homogenous microstructure [8,19,27].

According to the above conclusion, the sintered billets with an external diameter of 52 mm and an internal diameter of 20.8 mm were prepared as shown in Figure 9e. In particular, the bottom thickness was selected as 7 mm and BN powder was adopted as the filler material. Then the sintered billets were subjected to hot extrusion at 1100 °C with an extrusion ratio of about 10.56. Finally, the as-extruded tubes were reshaped at 950 °C. The finished production of TiBw/Ti6Al4V composite tubes is shown in Figure 9g. It can be seen from the transverse sections (Figure 9h) that the quasi-continuous structure remained despite the severe plastic deformation and TiBw became more disperse. In addition, the grain size of the Ti6Al4V matrix and the original β phase were considerably refined and the average width of the α phase decreased. As observed from the longitudinal section in Figure 9i, TiBw were rotated to align along the extrusion direction. Compared with the random distribution in the sintered microstructure, TiBw with directional alignment could play a better strengthening effect role in the aligned orientation. In addition, the oxygen content shown in Table 4 also suggested the better high-temperature strength. Some fragmented TiBw within the as-extruded microstructure could be observed leading to the decrease of their aspect ratios.

**Figure 10:**
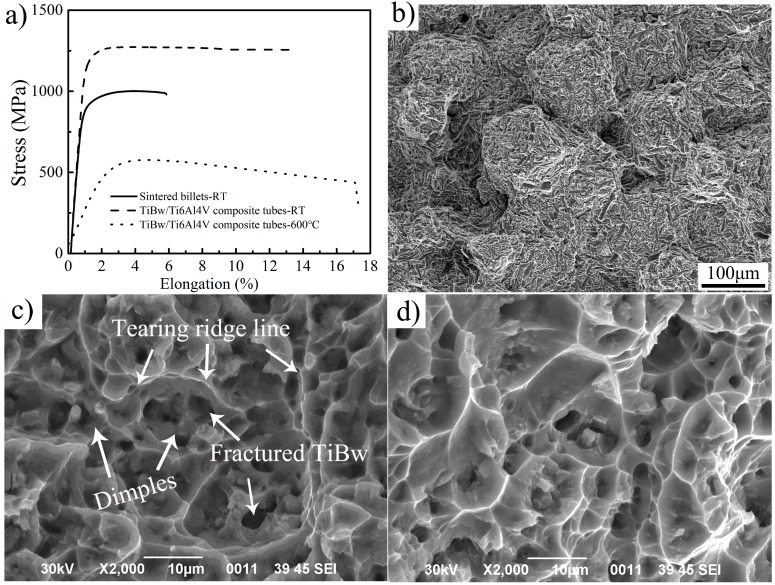
The tensile curves and fracture morphology of the TiBw/Ti6Al4V composite tubes: (**a**) Tensile curves at different conditions; (**b**) Fracture morphology of sintered billets at room temperature; (**c**) Fracture morphology of composite tube at room temperature; (**d**) Fracture morphology of composite tube at 600 °C.

Figure 10 shows the tensile properties and fracture surface of the TiBw/Ti6Al4V composite tubes. The tensile strength and elongation of the composite tubes at room temperature could reach 1240 MPa and 13.5%, respectively. Compared with those of the as-sintered composite, the tensile strength and elongation were increased by 16.2% and 81.5%, respectively. The main reasons for this significant improvement of ductility were as follows: (a) the alignment distribution of the TiB whisker reinforcement; (b) the refined grain size of the matrix. In the research of Gungor [28], β extrusion was used to produce seamless Ti6Al4V tubes with an ultimate tensile strength of 960 MPa, a yield strength of 857 MPa and an elongation of about 13%. In terms of the tensile properties at high temperature, the ultimate tensile strength was about 577 MPa at 600 °C, which was nearly 50 MPa higher than that of the extruded Ti6Al4V [29]. These comparisons demonstrate the superior strengthening effect of the quasi-continuous TiBw both at room temperature and high temperature. Meanwhile, the elongation for the extruded tubes increased from 14.7% at ambient temperature to 17.1% at 600 °C.

It can be seen from Figure 10b that the prior cracks seem to initiate and propagate along the network boundary of the sintered composites at ambient temperature, which might be referred to as intercrystalline fracture, which was consistent with the inferior ductility in Figure 10a. In contrast, plenty of dimples and tearing ridges, which were known as ductile fracture features, were observed at room temperature and 600 °C are shown in Figure 10c,d. The broken TiBw means there is strong interfacial bonding between it and the matrix.

## 4. Conclusions

In this paper, a novel extrusion for fabricating TiBw/Ti6Al4V composite tubes was proposed. It can fabricate the length-diameter ratio of a thin tube of difficult-to-deform materials. The numerical simulation was adopted to explore the relationship between the frictional coefficient, thickness, flow stress and shape of the extruded tubes. Moreover, the microstructure and mechanical properties of TiBw/Ti6Al4V composite tubes were also investigated. The conclusions are drawn as follows:

1. It is feasible to fabricate the TiBw/Ti6Al4V composite tubes via hot extrusion with a filler material.
2. Based on the simulation results, the deformation resistance of the filler material is relatively unimportant for the tube shape compared with the friction coefficient and the bottom thickness. Considering the difficulty of removing the filler material, BN powder can be adopted. In addition, with increasing the friction coefficient, inhomogeneous deformation at the head region tends to weaken. The cracking at the head region can be avoided by the coordination of the friction coefficient and the bottom thickness.
3. After extrusion, the transverse section microstructure of the TiBw/Ti6Al4V composite tube remained in the quasi-continuous structure and the TiBw were rotated to align along the extrusion direction. Moreover, the tensile strength and elongation can reach 1240 MPa, 13.5% at room temperature and 577 MPa, 17.1% at 600 °C, which result from dynamic recrystallization and TiB whisker strengthening.

## Figures and Tables

**Table 1:** Chemical composition (wt. %) of Ti6Al4V powders.

Al	V	Fe	Si	O	C	N	H	Ti
6.42	4.12	0.18	0.0.24	0.12	0.013	0.011	0.004	Bal.

**Table 2:** Material parameters of the TiBw/Ti6Al4V composite for finite element modeling.

Flow Stress *σ*	Frictional Coefficient *f*	Bottom Thickness *t* (mm)	Heat Capacity N/mm^2^ °C	Thermal Conductivity W·(m·K)^−1^	Poisson Ratio	Elastic Modulus GPa
0.5*σ*, 0.75*σ*, *σ*, 1.25*σ*, 1.5*σ*	0.7	3	7	2.4	0.3	130
*σ*	0.1, 0.2, 0.3, 0.5, 0.7	3				
*σ*	0.7	3, 6, 7, 8, 9				

**Table 3:** The simulation results of the extrusion process with different parameters (×: Cracked, √: Uncracked).

Bottom Thickness	*f* = 0.1	*f* = 0.2	*f* = 0.3	*f* > 0.3
3 mm	×	×	√	√
4 mm	×	√	√	√
5 mm	×	√	√	√
6 mm	√	√	√	√
7 mm	√	√	√	√

**Table 4:** The oxygen content of different stages (wt. %).

Titanium Powders	Milling Powders	Extruded Tubes
0.12	0.15	0.23
